# A Long-Term Study of the Biological Properties of ASF Virus Isolates Originating from Various Regions of the Russian Federation in 2013–2018

**DOI:** 10.3390/vetsci6040099

**Published:** 2019-12-06

**Authors:** Andrei Pershin, Ivan Shevchenko, Alexey Igolkin, Ivan Zhukov, Ali Mazloum, Elena Aronova, Natalia Vlasova, Alexander Shevtsov

**Affiliations:** Federal Center for Animal Health (FGBI ARRIAH), 600901 Vladimir, Russia; shevchenko@arriah.ru (I.S.); igolkin_as@arriah.ru (A.I.); zhukov@arriah.ru (I.Z.); ali.mazloum6@gmail.com (A.M.); aronova@arriah.ru (E.A.); vlasova_nn@arriah.ru (N.V.); shevcov@arriah.ru (A.S.)

**Keywords:** ASF, epidemiology, experimental infection

## Abstract

Biological properties of the African swine fever (ASF) virus isolates originating from various regions of the Russian Federation (2013–2018) were studied in a series of experimental infections. Comparative analysis allowed us to establish the differences in the key characteristics of the infection, such us the duration of the incubation periods, disease, and the onset of death. The incubation period averaged 4.1 days, varying from 1 to 13 days. An average duration of the disease was 6.3 days and varied from 0 to 18 days. Overall case fatality was 94.5%, and antibodies were detected only in 19.3% of the animals. The biological properties of isolates Odintsovo 02/14 and Lipetsk 12/16 were significantly different from others. For this two, the presence of antibodies to the virus was detected in 71.4% and 75% of animals respectively and mortality levels were of 87.5% and 50%.

## 1. Introduction

African swine fever (ASF) is a contagious viral disease of pigs and wild boar. It typically manifestoes as a hemorrhagic fever, but can also occur in various forms from hyperacute to inapparent.

The case fatality rate of susceptible livestock reaches 100% for highly virulent isolates. To date, there are no effective specific prevention measures nor treatment for ASF [1,2,3]. The control strategy for ASF is aimed at compliance with the requirements of biosafety, as well as rapid diagnosis, followed by culling of infected animals and decontamination.

An animal is usually infected either by the alimentary route (eating food waste and raw materials from infected animals; contaminated feeds) or when in contact with infected animals and contaminated objects. Infected soft ticks of the Ornithodoros genus can also spread infection. The important role of O. erraticus in disease maintenance was clearly confirmed in Portugal and Spain [4]. However, these ticks are believed to be absent in the parts of EU currently affected by the epidemic [5].

African swine fever virus genotype I was first introduced into Europe in 1957 in Portugal. A second introduction of the same genotype occurred in 1960 again in Portugal. Virus spread through the Iberian Peninsula, where ASFV persisted for more than 30 years with several escapes to both American and European countries. All these outbreaks were efficiently controlled, except on the island of Sardinia [4]. ASF virus genotype II was introduced to the Eurasian continent in 2007. And its control is currently very challenging in most of the affected countries.

The causative agent of ASF is a double-stranded DNA virus of the Asfarviridae family. Virulent isolates usually have hemadsorbing activity and ASFV genome may undergo some changes [6,7,8].

Since then, changes in the genetic structure of the virus were found repeatedly in isolates from various regions of Russia and European countries [7,8,9]. However, available ASFV whole-genome sequences show the stability of the ASFV genome within of the modern European genotype II [10]. Nevertheless, a detailed study of circulating isolates requires both molecular genetic studies and examination of its virus’s biological properties in an experimental infection. The most significant epizootological indicators are: duration of incubation period, disease, and the onset of death for pigs. The information regarding seroconversion levels is necessary for selection of diagnostic techniques.

Since 2007, ASF persists in Russia. It causes massive economic losses because of the high costs of slaughter of animals and the elimination of outbreaks, as well collateral losses caused by the restrictions imposed on domestic and international trade [11,12].

This paper summarizes the results of 15 experimental infections of pigs with various Russian isolates of the ASF in 2013–2018.

## 2. Materials and Methods

### 2.1. Experimental Challenge

One hundred forty three pigs were challenged with 15 ASF virus isolates. Experimental challenges were carried out according to the “1 isolate–1 experiment–1 box” scheme, using different doses and routes of infection. We used 181 piglets weighing 15–25 kg, obtained from conventional pig farms. One hundred forty three pigs were inoculated with the virus and 38 were used as direct contact (Table 1) in 12 out of 15 challenges. The animals were stationed in the isolated boxes of the vivarium facility of the FGBI “ARRIAH” (BSL 3). Animals were kept quarantine for 5–10 days. At the same time the sera samples were examined to confirm the seronegative status for the main infectious diseases of pigs (ASF, CSF, etc.). The feeding and housing conditions complied with the standards for animals of the age groups used. The infectious dose and using of control animals were determinate according to the needs of the current experimental challenge.

### 2.2. Infectious Material

**Figure 1:**
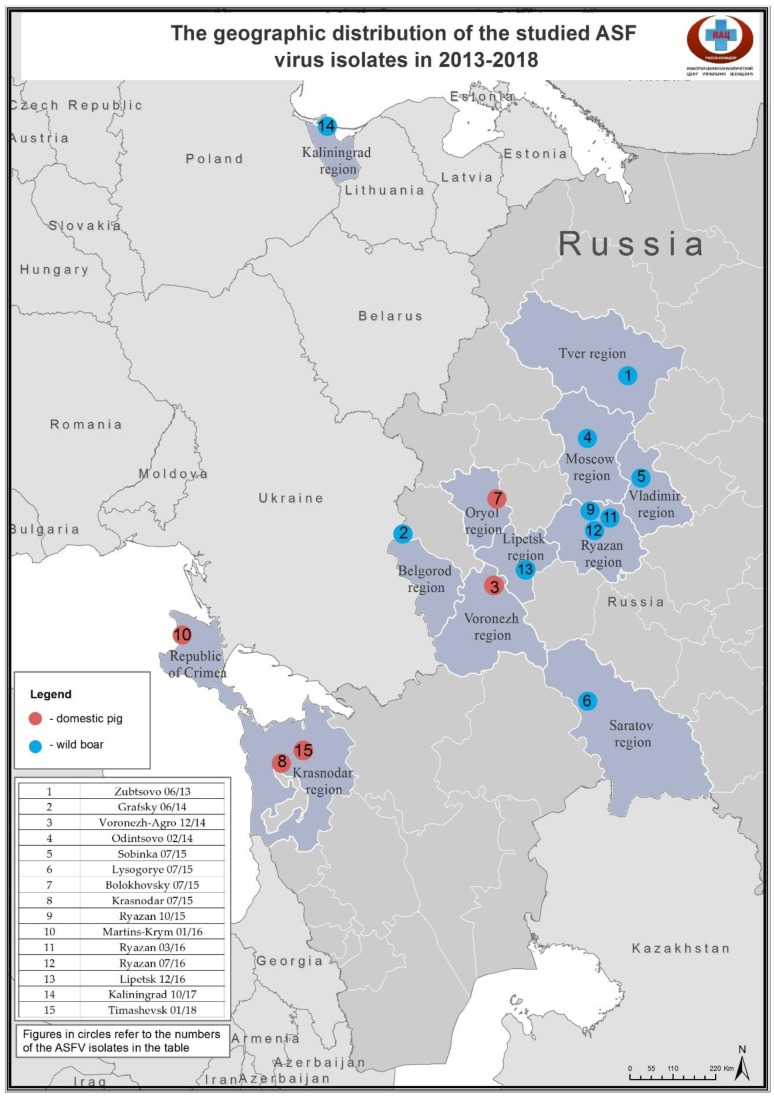
The geographic distribution of the studied African swine fever (ASF) virus isolates in 2013–2018.

In 2013–2018, ASF outbreaks were officially notified in 38 regions of the RF. In this study, we used isolates from 12 different regions, selected by cultural properties and spatial-temporal characteristics, as well as various doses and routes of infection (Figure 1, Table 1). Pathological material originated from wild boars (9 isolates) and domestic pigs (5 isolates). Virus containing materials were cultivated in the porcine bone marrow cell cultures (PBM CC) for 1 passage. The Odintsovo 02/14 isolate originates from a wild boar, shot on the territory of the Tarakanovsky forestry of the Odintsovo district, Moscow region. Isolate was passaged in PBM CC for three consecutive passages [13]. One of the viruses was isolated from feces of a wild board of the Lipetsk region. Some of the isolates have been sequenced and published [9,14]. Whole-genome sequence of Odintsovo 02/14 is available in GenBank (KP843857). Central variable regions of some isolates have been compared [15]. But for most isolates eventual genetic variations are unknown.

A 10% suspension of the spleen (or another tissue in some cases) was prepared in a sterile saline solution and diluted to the required infecting dose. The design of individual experiments included intact animals in direct contact with the infected pigs to simulate the natural viral transmission. Rectal body temperature and clinical signs of animals were observed daily. Blood samples were taken from the pectoral vascular plexus using vacuum tubes and steel needles. 

### 2.3. Diagnostic Techniques

Enzyme-linked immunosorbent assay (ELISA) was performed using the Ingezim PPA Compac K3 kit (Ingenasa, Madrid, Spain) in accordance with the manufacturer’s instructions.

Immunoperoxidase test (IPT) was performed in accordance with the standard operating procedure by CISA-INIA (SOP/CISA/ASF/IPT/1). IPT was used as the confirmatory test on the early stages of infection.

Real time polymerase chain reaction (RT PCR) was performed using the test system by FGBI “ARRIAH.”

This study was approved by the Institutional Animal Care and Use Committee (IACUC) of FGBI “ARRIAH” (project ID is GJ 3.1.2013.2018), and conducted in compliance with the local and federal guidelines.

## 3. Results

In this study, the incubation period ranged from 1 to 13 days with an average of 4.1 (Table 1). The inoculation day was considered day 0. The length of an incubation period was measured as time between Day 0 and the first day of rectal temperature >40.5 °C in any infected animal. The incubation periods for the remaining animals were not estimated because of the possible contact infection.

For 10 out of 12 isolates incubation periods varied from 2 to 5 days. For isolates Zubtsovo 06/13 and Grafsky 06/14 inoculated at a dose of 1 HAU, incubation periods were 13 and 12 days, respectively. At a dose of 10 HAU incubation period was 5 days.

Duration of the disease was defined as the time between the appearance of hyperthermia and death or recovery. It ranged from 0 to 18 days, with an average of 6.3 days (Table 1). Clinical signs (including hyperthermia and weakness) were absent is some animals, despite the presence of viral genome and antibodies in its blood.

Necropsy of dead pigs revealed characteristic pathomorphological signs of ASF: splenomegaly, hyperplasia, and hyperemia of regional lymph nodes, hemorrhages in kidneys, pulmonary edema, and other typical lesions have been revealed like described before [16]. The degree of manifestation was depended on the time of sickness before death. The longer the animal stayed alive the severe the pathomorphological signs were.

Ingenasa ELISA kits were used to detect seroconversion. It was detected in 35 pigs or 19.3%.

Eight of the 181 (4.42 % total or 22.85% of seropositive animals) animals survived infection. No clinical signs of ASF were found in 30 days (isolates Lipetsk 12/16, Bolokhovsky 07/15, Lysogorye 07/15, Odintsovo 02/14). For Bolokhovsky 07/15 isolate, animals without clinical signs of ASF were slaughtered on day 16 post infection. A necropsy did not indicate characteristic pathoanatomical signs of ASF. In this paper, these animals are considered as recovered.

The probability of death after infection for animals with a detectable level of antibodies was 77.15%, with a total mortality rate of 94.5%.

The mortality rate for pigs on the 35th day post infection with Lipetsk 12/16 isolate was 50%. On this day, surviving animals were re-infected with the homologous highly virulent isolate Orlovsky 03/17 at a dose of 1000 HAU. The animals showed no clinical signs of ASF for 30 days after re-infection. Antibodies were present in the serum of animals throughout the experiment. On day 56, all the animals were slaughtered. A necropsy did not indicate characteristic pathoanatomical signs of ASF, except for a slight increase of submandibular and mesenteric lymph nodes.

Dynamics of the antibody production in pigs are showed in Table 2.

The biological properties of the isolates Odintsovo 02/14 and Lipetsk 12/16 significantly differed from the other isolates of 2013–2018. For said isolates antibodies were found in the sera of 10 out of 14 infected animals (71.4%) and 6 out of 8 (75 %), respectively, with mortality rates of 87.5% and 50%. For isolate Grafsky 06/14 antibodies were found in 1 animal out of 16 (6.25%), and for Ryazan 07/16 isolate no antibodies to the ASF virus were found.

The probability of antibody detection in animals infected by contact was higher than during the initial direct infection. In our studies, antibodies were detected in 14 out of 38 contact animals (36.84%) and in 21 out of 143 inoculated animals (14.68%).

Seven experiments were carried out using different infecting doses (0.1–10 HAU). In most cases, antibodies were detected in groups of animals infected with the lowest dose, except for isolates Voronezh-Agro 12/14 (10 HAU) and Ryazan 10/15 (10 HAU), where antibodies were detected in both groups (Table 3).

**Figure 2:**
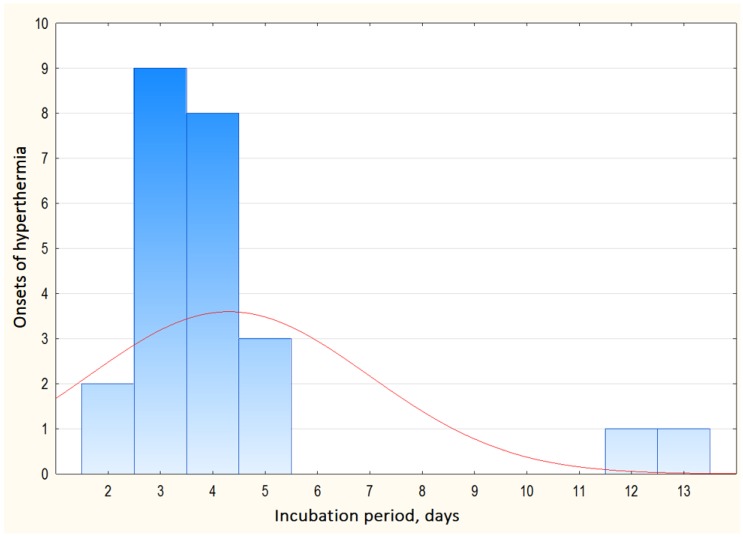
Distribution of the duration of incubation periods for studied isolates (first case of hyperthermia in any of the infected pigs).

Antlibodies were present in blood of some pigs. They were usually detected at 9–14 d.p.i., usually a few days before the death of the animal (Figure 2). In contact animals, antibodies were produced at 15–20 days after beginning of the experiment. It indicates the rapid replication of the virus in inoculated animals and the onset of virus shredding. The formation of immunoglobulins in a detectable amount happened in the least “physiological time” (7–9 days).

For isolate Lipetsk 12/16 we found an interesting difference in the production of antibodies in animals. In one pig antibodies were detected from day 10. This pig died on day 14. In the three surviving pigs, antibodies were detected later, days 13 to 17. Antibody titers were relatively high (more than 1:10). One pig had a subclinical form of the disease. In this pig, antibodies were detected between 13 to 25 days after infection by the immunoperoxidase test only. In the ELISA the result was doubtfull.

**Figure 3:**
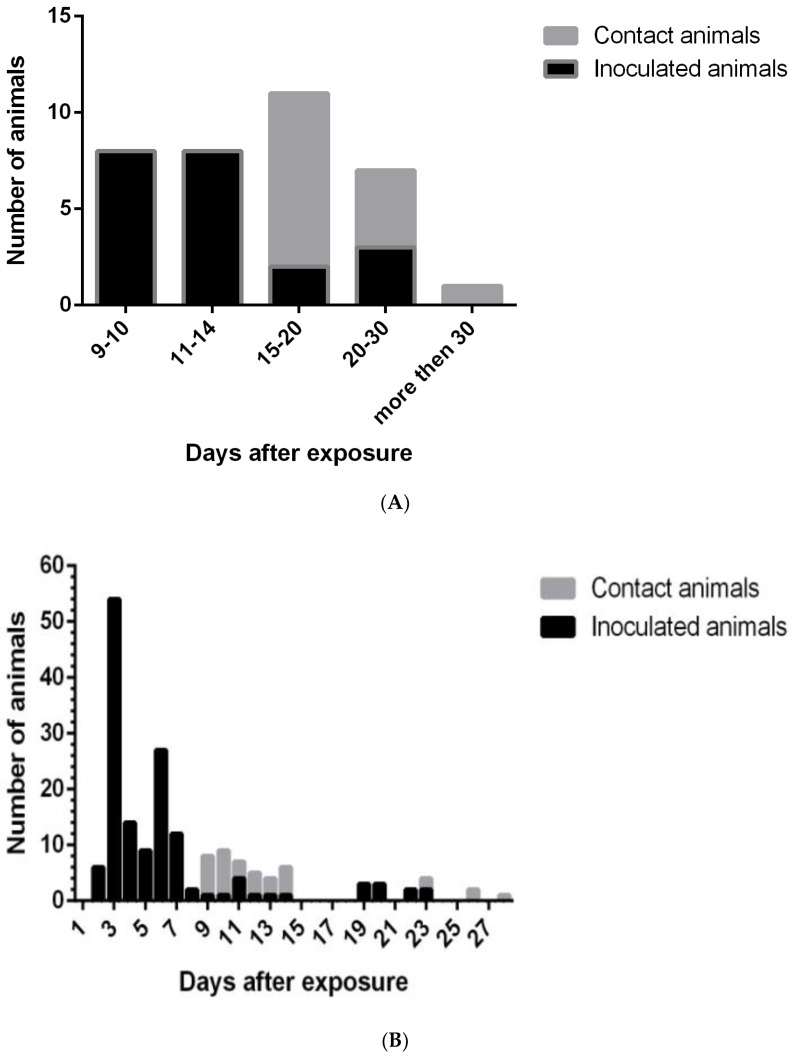
The temporal distribution of seropositive (**A**) and PCR-positive (**B**) animals.

The temporal distribution of PCR-positive animals is shown in the Figure 3. It indicates the first day when animal was tested PCR-positive. It is important to keep in mind that samples were not taken daily. After first postitive result all animals remained positive till the death exept for surviving animals from experiments with Odintsovo 02/14, Lipetsk 12/16, and Timashevsk 01/18 isolates where animals were PCR negative in the end of the observation period.

## 4. Discussion

The biological properties of the selected ASF isolates were found to be different. It included duration of the incubation periods, disease, and the time point of death in both infected and contact animals. We also observed the asymptomatic disease, as well as the recovery after infection.

For isolates Zubtsovo 06/13 and Grafsky 06/14 we have seen a dose-dependent effect. At a higher dose of 10 HAU incubation period was two times higher compare to 1 HAU.

Dose dependence has also been demonstrated by Howen et al., where increased inoculation dose of ASFV was associated with a significant decrease in survival duration of the pigs [17].

In the study with the ASFV Malta’78 isolate different groups showed more variable outcomes. In the group with high dose inoculation, only one directly inoculated animal succumbed to infection while the others survived until the end of the trial. In the group with the lower inoculation dose, all inoculated animals survived while five out of seven animals commingled with the experimentally inoculated pigs died [18].

For other isolates we did not seen any clear dose-dependent effect. For example in Voronezh-Agro 12/14 experiments some animals infected with higher dose died after low dose animals.

According to previous research, incubation periods during ASF epizootics varied greatly. On the Iberian Peninsula (Spain) in 1957–1995, they ranged from 4–6 days with acute form of the disease and from 6–8 days with subacute, or even up 20 days [19]. During ASF outbreaks in France, incubation period ranged from 2–4 days, with a 10 days maximum. You can refer to the article by V.V. Kurinnov for additional information [20].

The authors showed that all ASFV isolates from 2007–2015 had a high virulence and contagiousness levels, regardless of the time and route of infection (for both domestic and wild boar). A formula for the maximum probability of an incubation period for ASF has been established based on the statistical analyzes. It includes a central trend—the median and the minimum-maximum value of the total quantile distribution of 4 (2–10) days [20].

In our study the incubation period for various isolates averaged 4.1 days with extreme values from 1 to 13 days, the duration of the disease averaged 6.3 days and varied from 0 to 18 days, the overall case fatality rate was 94.5%, antibodies were detected in 19.3% of the animals. The probability of detecting antibodies in animals infected by contact (“natural model”) is higher than that of the initially infected.

The share of seropositive animals remained low regardless of the isolation year. Thus, the presense of antibodies in pigs is associated with the biological properties of specific isolates or immune system of infected animal. Some animals (isolate Lipetsk 12/16) survived witout any clinical or pathological signs after re-infection with highly virulent isolate. Therefore, the surviving animals had a protective immunity after first challenge. Not surprisingly, the production of antibodies did not significantly affect the survival rate of the animals in experimental challenges with Russian ASF isolates 2013–2018. Seroconversion at an early stage post infection was not linked with an increased chance of survival, which is consistent with the previous results [21]. However, the surviving animals had a different course of the disease. For isolates Lysogorie 07/15, Bolokhovsky 07/15, Timashevsk 01/18, surviving animals suffered a prolonged fever, accompanied by weakness and refusal of food. The biological properties of isolates Lipetsk 12/16 and Odintsovo 02/14 were strikingly different. It was characterized by the absence or just a slight manifestation of clinical signs in infected pigs.

First days antibodies were usually detected between 9–14 d.p.i. for inoctulated animals and between 15–30 days for contact animals. The initial detection of antibodies in contact and infected animals more than 20 days after inoculation may indicate a slow pace of the infection. It is also possible that the animals were not infected during the initial introduction of virus, but infected subsequently upon contact with the infected animals. A long time can pass from the viral entry into a pig population until the onset of seroconversion in secondarily infected animals. This is important to consider when choosing a diagnosing technique for ASF.

Since the onset of the epizootic in the Russian Federation antibodies have been detected in ASF affected animals including wild boars and domestic pigs in both primary and secondary outbreaks [22]. However, serodiagnostic techniques are currently less effective than detection of viral genome or antigen. Because of the high virulence of ASF virus, its circulation in the Russian Federation was accompanied by low seroprevalence [23,24,25], which is confirmed by the results of research, presented in this article. A large number of serological studies are conducted annually in the Russian Federation, aiming for the detection of antibodies. Those studies are low-performing. In 2011–2017, 173,000 samples were tested for presence of antibodies to the ASF virus as part of the national epidemiological monitoring. Only 31 samples tested positive [26]. As vaccines against ASF do not yet exist, the results obtained by direct and indirect methods are equivalent for the laboratory diagnosis of the disease. However, both methods should be used in accordance with the limits of their applicability.

A significant increase in the detection of seropositive samples was observed in the Baltic countries in 2015 in sera of wild boars. It more than doubled the results of the previous year [26]. A substantial part of the pigs had antibodies after infection with Lipetsk 12/16 isolate. Interestingly, this isolate has been collected from feces of wild boar. We believe that changes in biological structure of this isolate have occurred after passaging through an animal (a boar) but it has to be proved by genetic research.

The results indicate that ASFV isolates circulating in the Russian Federation can cause an asymptomatic disease with reduced mortality (50% mortality for Lipetsk isolate 12/16).

Reduced mortality and an increasing number of asymptomatic and chronic cases in endemic areas will require a different approach to laboratory diagnosis of ASF. Ståhl et al. found no evidence in the papers of any significant role in the epidemiology of the disease of survivors or seropositive animals. As demonstrated in several studies, levels of viraemia and virus shedding decrease after the acute phase [27]. Such survivors can be untraceable with direct methods, aimed for the detection of the antigen or virus, complicating the estimation of distribution of the disease. Additionally, the presence of antibodies is a good marker of subacute or chronic infection. Parallel testing of samples by direct and indirect methods should become mandatory.

According our personal observations, in Russian Federation, in case of ASF outbreak, necropsy of dead animals is often performed only in form of taking samples for laboratory tests, dead animals are destroyed as soon as possible, as factors of biological danger, especially on the big farms. Also, necropsy may be done only for the first dead animal. Sometimes interpretations of the findings by the different veterinarians are not clear enough for comparison. Experimental challenge should become one of the main methods for examination of the biological properties of new isolates because it makes it possible to study the clinical and pathological signs of the disease, which may not be possible in the event of an ASF outbreak. It should be noted that the data obtained during of experimental challenge might differ from the field conditions.

For example, the isolate Timashevsk 01/18 did not cause the clinical signs of the acute form of the disease the field conditions. There was also a large number of seropositive pigs detected, so it was assumed that a virus of reduced virulence caused the outbreak. One hundred samples were taken from animals expressing no clinical signs of ASF or atypical signs. Viral DNA was detected in 73 samples, while 37 samples considered doubtful in PCR, 36 samples were also tested positive for antibodies to ASF virus [28]. However, during the experimental challenge, the mortality rate was 90%. Clinical signs were characteristic of the acute form of ASF.

This difference can be caused by a non-coincident physiological state and age of the animals. In the study by J. Post et al. the age of the animals influenced the presence of cytokines (TNF-α, IL-12, and IL-10). At the same time, surviving animals had a significantly higher level of IL-10 in the serum at the time of infection [29]. We did not find any significant effect of the level of IL-10 in the serum before infection on the survival of animals [30]. It also imposes restrictions on the possibility of comparing the biological properties of isolates by comparing the results of experimental infections and the field conditions.

In addition, invasive methods of blood sampling are likely to accelerate the onset of animal death, given the development of coagulopathy and hemorrhagic syndrome in ASF, especially for highly virulent isolates.

The route of infection may also influence the course of the disease [17]. Intramuscular infection is optimal in terms of delivery and dosage of the virus under laboratory conditions. However, this does not reproduce the mechanism of infection in pig farms.

## 5. Conclusions

We found differences in the incubation period, the clinical course, and the survival time of the inoculated and contact animals. The differences described above may indicate the heterogeneity of the viral population of the studied isolates of the ASF virus. The differences may also be within the biological range of the disease as long as there is no proof of genetic variance in the tested isolate. The biological properties of the isolates Odintsovo 02/14 and Lipetsk 12/16 significantly differed from the other isolates of 2013–2018. For said isolates, antibodies were found in 71.4% and 75% of animals, respectively, with mortality rates of 87.5% and 50%.

ASF-specific antibodies were detected in a limited number of animals. Therefore, the detection of antibodies for the purpose of early detection of the disease is not advisable. It is also not efficient for determining the health status of animals in surveillance programs. However, given the possibility of ASF re-infection, it is necessary to conduct parallel studies with direct methods to identify antibodies in livestock lacking constant control from the veterinary services in wild boars, as well as in large pig-breeding enterprises, where the so-called natural technological waste (the amount of pigs that are dying regularly due to principles and quality of technological system) can mask soft or asymptomatic course of infection.

## Figures and Tables

**Table 1:** Comparison of incubation periods, duration of the disease, amount of seropositive animals, and the onset of death.

Isolate and Dose		Animals		Duration of the Disease, Days	Seropositive Animals	Death of Animals		Incubation Periods	
		Inoculated	Contact			%	Days	Fever, Day	PCR *
Zubtsovo 06/13	10 HAU ^1^	5	2	5–9	0	100	10–21	5	0, **4**
Zubtsovo 06/13	1 HAU	6	2	5–11	4	100	21–33	13	10, **14**
Grafsky 06/14	10 HAU	6	2	5–10	0	100	11–16	5	3, **5**
Grafsky 06/14	1 HAU	6	2	6–10	1	100	18–33	12	8, **11**
Voronezh-Agro 12/14	10 HAU	6	0	5–11	1	100	8–14	3	0, **3**
Voronezh-Agro 12/14	1 HAU	6	0	3–8	0	100	7–12	4	0, **3**
Voronezh-Agro 12/14	0.1 HAU	6	0	2–5	1	100	6–10	4	0, **3**
Odintsovo 02/14	10 HAU i/m	5	2	0–18	5	87.5	after 10	2	0, **3**
Odintsovo 02/14	50 HAU i/n	5	2	1–11	5	87.5	after 10	4	0, **3**
Sobinka 07/15	10 HAU	6	2	6–14	0	100	10–21	4	0, **3**
Sobinka 07/15	1 HAU	6	2	4–15	2	100	8–32	4	0, **3**
Lysogorye 07/15	1 HAU	6	2	3–11	0	100	8–16	2	1, **3**
Lysogorye 07/15	0.1 HAU	6	2	5–10	1	87.5	after 9	5	4, **7**
Krasnodar 07/15	10 HAU	6	2	2–5	0	100	7–16	4	3, **6**
Krasnodar 07/15	1 HAU	6	2	3–8	1	100	7–17	3	0, **3**
Ryazan 10/15	10 HAU	6	2	6–11	2	100	9–23	3	0, **3**
Ryazan 10/15	1 HAU	6	2	4–8	0	100	9–15	3	0, **3**
Bolokhovsky 07/15	10 HAU	6	2	5–13	3	75	after 9	3	0, **3**
Martins-Krym 01/16	50 HAU	4	2	5–11	1	100	9–18	3	0, **3**
Ryazan 03/16	10 HAU	8	0	3–7	1	100	8–13	3	0, **3**
Ryazan 07/16	10 HAU	6	2	3–7	0	100	8–19	4	0, **3**
Lipetsk 12/16	1000 HAU	6	2	0–8	6	50	after 8	4	0, **3**
Kaliningrad 10/17,	10 HAU	6	0	2–10	0	100	10–20	3	0, **3**
Timashevsk 01/18	10 HAU	8	2	5–19	1	90	after 7	3	0, **2**
Total/Average		143	38	6.30	35	94.5		4125	

^1^ HAU—hemadsorbing units. * Bold numbers indicate the beginning of positive results for ASFV genome detection (first positive animal in the group), non-bold numbers indicate the previous collection day.

**Table 2:** The percentage of seropositive animals infected with ASF virus isolates isolated in 2013–2018.

Year	Pigs Infected	Isolates Tested	Seropositive		Survived
			Pigs	%	
2013	15	1	4	26.7	0
2014	48	3	13	27	2
2015	70	5	9	12.9	3
2016	32	4	8	25	4
2017	6	1	0	0	0
2018	10	1	1	10	1
Total	181	15	35	19.3	10

**Table 3:** The dynamics of the detection of seropositive animals during experimental infection.

Isolate	Dose	Route	Blood Sampling Days *	Livespan, Days
Isolates 2013				
Zubtsovo 06/13	1 HAU	Cont	3, 7, 10, 14, 18, 21, 24, **28, 30**	31
		I/m	3, 7, 10, 14, 18, 21, 24, **28**	29
		I/m	3, 7, 10, 14, 18, 21, 24, 28, **30**	31
		I/m	3, 7, 10, 14, **18, 21**	22
Isolates 2014				
Grafsky 06/14	1 HAU	Cont	3, 5, 8, 11, 15, 19, 22, 26, 29, **32**	33
Voronezh-Agro 12/14	10 HAU	I/m	3, 5, 9, **12**	14
	0.1 HAU	I/m	3, 5, **9**	10
Odintsovo 02/14	10 HAU	I/m	3, 6, **9**	11
		I/m	3, 6, **9, 12, 15, 18, 21, 24, 28, 32**	no **
		I/m	3, 6, **9, 12, 15**	18
		Cont	3, 6, 9, 12, 15, **18, 21, 24, 28**	30
		Cont	3, 6, 9, 12, **15, 18**	19
Odintsovo 02/14	50 HAU	I/n	3, 6, 9, **12, 15, 18, 21, 24, 28, 32**	no **
		I/n	3, 6, 9, **12**	14
		I/n	3, 6, 9, **12, 15**	16
		Cont	3, 6, 9, 12, 15, **18**	21
		Cont	3, 6, 9, 12, 15, 18, **21**	22
Isolates 2015				
Krasnodar 07/15	1 HAU	Cont	3, 6, 9, 12, **15**	17
Ryazan 10/15	10 HAU	I/m	3, 6, **9**	13
		Cont	3, 6, 9, 12, 15, **19, 22**	24
Sobinka 07/15	1 HAU	Cont	3, 5, 9, 13, 16, 19, 23, 27, **30**	31
		Cont	3, 5, 9, 13, 16, 19, 23, **27**	28
Lysogorye 07/15	0.1 HAU	Cont	4, 7, 11, 15, **18, 22, 28**	no **
Bolokhovsky 07/15	10 HAU	I/m	3, 6, 9, **14, 16**	no **
		I/m	3, 6, **9**	10
		Cont	3, 6, 9, 14, **16**	no **
Isolates 2016				
Martins-Krym 01/16	50 HAU	I/m	3, 6, **10**	11
Ryazan 03/16	10 HAU	I/m	3, 6, 9, **12**	13
Lipetsk 12/16	1000 HAU	I/m	3, 6, 10, 13, 17, 20, 25, **28, 32, 35**…	no **
		I/m	3, 6, 10, 13, **17, 20, 25, 28, 32, 35…**	no **
		Cont	3, 6, 10, 13, **17**	18
		I/m	3, 6, **10, 13**	14
		Cont	3, 6, 10, 13, **17, 20, 25, 28, 32, 35…**	no **
		I/m	3, 6, 10, **13, 17, 20, 25, 28, 32, 35…**	no **
Isolates 2018				
Timashevsk 01/18	10 HAU	I/m	2, 5, 8, 11, **15, 18, 22, 25, 37**	no **

I/m—intramuscular; I/n—intranasal; cont—contact; * bold numbers indicate the beginning of positive results for ASFV antibodies; ** animals were slaughtered.
